# Household Transmission of Zoonotic Influenza Viruses in a Cohort of Egyptian Poultry Growers

**DOI:** 10.2196/resprot.4331

**Published:** 2015-06-22

**Authors:** Amira S El Rifay, Mona A Elabd, Dina Abu Zeid, Mokhtar R Gomaa, Li Tang, Pamela P McKenzie, Richard J Webby, Mohamed A Ali, Ghazi Kayali

**Affiliations:** ^1^ Center of Excellence for Influenza Viruses National Research Centre Giza Egypt; ^2^ St Jude Children's Research Hospital Memphis, TN United States; ^3^ St Jude Children's Research Hospital Memphis, TN United States

**Keywords:** influenza, avian, epidemiology, cohort

## Abstract

**Background:**

The highly pathogenic avian influenza H5N1 viruses and the low pathogenic H9N2 viruses are enzootic in Egyptian poultry. Several cases of human infection with H5N1 were reported in Egypt. We previously determined that the seroprevalence of H5N1 antibodies in Egyptians exposed to poultry is 2.1% (15/708), suggesting that mild or subclinical infections with this virus occur. We aim to measure the incidence of avian influenza infection in Egyptians exposed to poultry, study risk factors of infection, study the resulting immune response, study household transmission rates, and characterize the viruses causing infections.

**Objective:**

The objective of the study is to design a 7-year, prospective, household-based cohort investigation to determine incidence and household transmission of avian influenza viruses in humans exposed to poultry.

**Methods:**

At baseline, we will collect sera to measure antibodies against influenza A. Field nurses will visit enrolled subjects at least weekly to check for influenza-like illness symptoms and verify influenza infection by a point of care rapid test. From subjects with influenza infection and their household contacts, we will collect nasal swabs, throat swabs, and nasal washes to characterize the antigenic and genetic makeup of influenza viruses infecting humans. The nurse will also obtain 2x 3-ml blood samples, one for serology, and another for isolating peripheral blood mononuclear cells.

**Results:**

Results from this cohort will enhance our understanding of the transmission of avian influenza viruses to humans in a country where such viruses are enzootic.

**Conclusions:**

This may enhance public health efforts aimed at reducing this burden.

## Introduction

### Epidemiology of Avian influenza

Several subtypes of influenza A virus adapted to poultry hosts from wild birds and some were able to cause infections in humans exposed to infected poultry [1,2]. Only two subtypes of influenza A, H3N2 and H1N1, currently circulate in humans. However, avian influenza viruses (AIV) of the H5, H7, and H9 subtypes are known to infect humans. H5N1 has caused more than 660 human infections since 2003, of whom, around 60% died [3]. Recently, an H7N9 virus emerged in China, where it continues to cause severe infections among humans [4]. H9N2 viruses are also capable of causing human infection, but cases are sporadic and the infection is not fatal [5,6]. In Egypt, H5N1 viruses are enzootic in poultry, and human cases have been reported continuously since 2006. The total number of cases reported so far in Egypt is 177, of whom, 63 died [3].

Our surveillance for AIV in Egyptian poultry since 2009 revealed that the threat of H5N1 viruses is widespread, as the virus was detected in all poultry production sectors (commercial farms, backyard poultry, live bird markets, abattoirs), in most poultry species, and throughout the year [7]. In early 2011, H9N2 viruses were detected in Egyptian poultry and were found to cocirculate with H5N1 viruses and frequently infect the same avian host [8-10]. H5N1 and H9N2 viruses in Egypt were found to be continuously evolving and contain several markers of adaptation to the human host [11-13].

Alongside our poultry surveillance, we designed and conducted a 3-year, prospective, controlled, seroepidemiological study that enrolled 750 poultry-exposed and 250 unexposed individuals in Egypt [14]. We found that, at baseline, the seroprevalence of anti-H5N1 antibodies (titers ≥ 80) among exposed individuals was (2.1%) 15/708, significantly higher than that among the controls (0%) 0/224. Having chronic lung disease was a significant risk factor for infection. In follow-up, seroprevalence was low (< 0.62%) < 4/649, and not statistically different between the two study groups. Antibodies against H9N2 were not detected at baseline when H9N2 was not circulating in poultry. At follow-up, H9N2 was detected in poultry, and consequently, the seroprevalence among exposed humans was between 5.9% (38/648) and 7.5% (51/682), statistically higher than that among the unexposed subjects. Vaccination of poultry, older age, and exposure to ducks were risk factors for H9N2 [15].

However, by design, seroepidemiological studies do not accurately measure incidence of infection or transmission rates, and do not allow us to characterize the AIV causing infections and the associated immune response. Hence, we plan to conduct a large prospective household study among Egyptian backyard poultry growers designed to study, in real time, AIV infections in this population.

### Study Objectives

This study has four primary objectives: (1) to estimate infection incidence of avian influenza (AI) in poultry-exposed human populations; (2) to estimate seroprevalence of AI in poultry-exposed human populations; (3) to investigate potential risk factors associated with AI infections in poultry-exposed individuals; and (4) to investigate secondary infection risk for household contacts.

The secondary objectives of this study are: (1) to characterize the antigenic and genetic makeup of AIV infecting humans; (2) to monitor the pathogenicity and disease severity of AIV causing human infections and the associated immune response; and (3) to investigate the serologic response following confirmed influenza virus infection.

The study design’s main feature is close monitoring (up to twice weekly) of the study subjects by trained medical personnel. This will allow us to detect influenza infections in real time, thus enabling timely collection of biological samples and disease prognosis data.

## Methods

### Study Population and Setting

**Figure 1:**
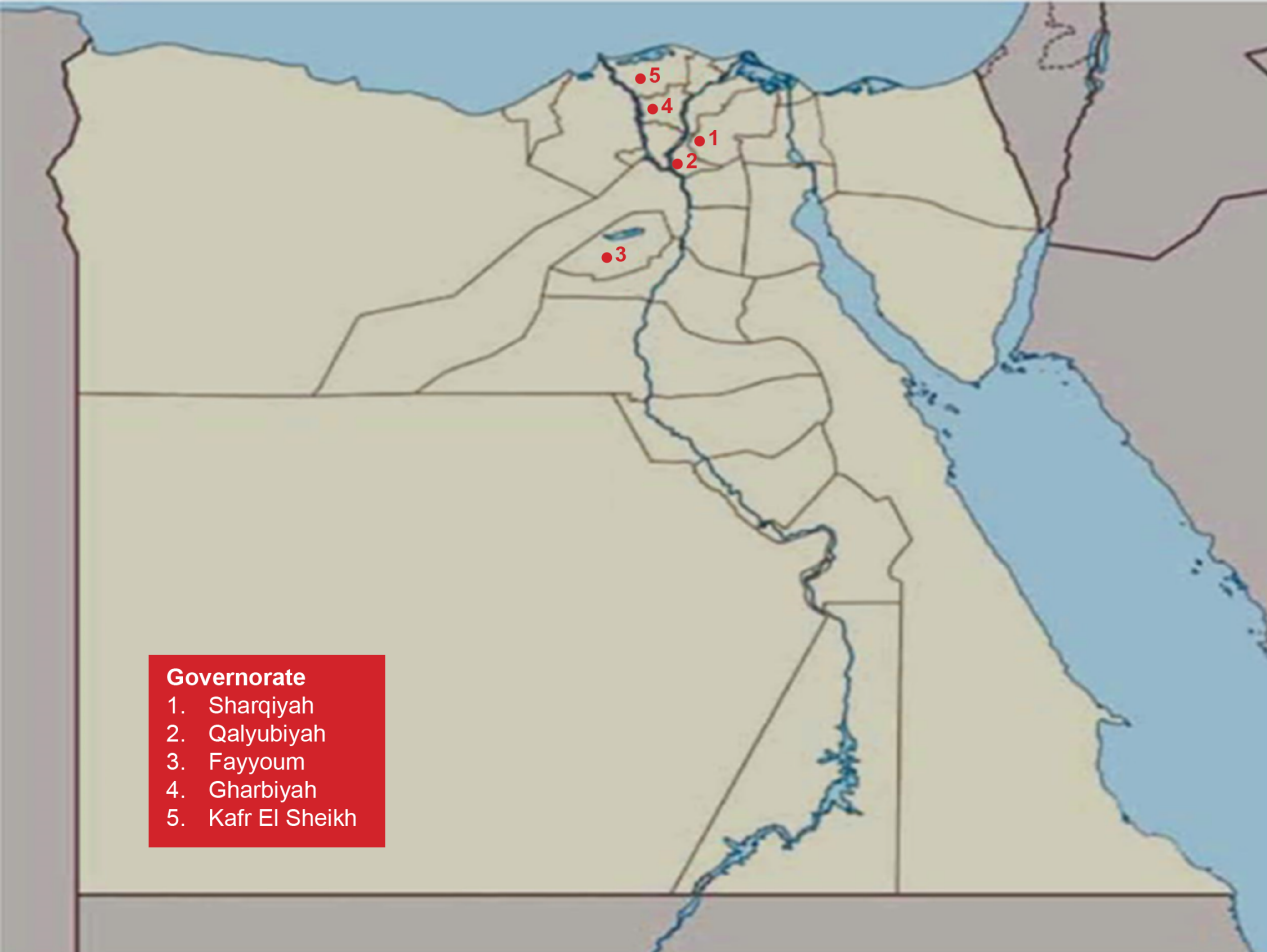
Map showing locations of the study sites.

We will enroll 240 households from the rural areas of Egypt at which backyard poultry is raised. We expect that the 240 households will yield a sample size of 2400 poultry exposed individuals. The majority of the reported human cases of H5N1 in Egypt were located in the Nile Delta region north to the capital Cairo. Our cohort of poultry-exposed subjects will be assembled from villages from 4 governorates in this area, as well as one governorate south of Cairo, where human cases of AIV were also reported (Figure 1 shows this). A village, in which we already conduct poultry surveillance for AIV, will be selected per governorate.

From each study site, we will enroll 48 households (480 individuals) exposed to poultry. We expect that our study population will include adults, children, males, females, and ethnic minorities, reflecting the distribution of these groups in the general Egyptian population.

Enrolling households that raise poultry will fulfill the sampling quota. Within the village, households will be randomly selected using the geospatial sampling capability of the R statistical software. All members of a selected household older than 2 years of age, and where poultry is raised, will be invited to participate and enrolled. Current data show that almost all H5N1 cases were in individuals older than 2 years, thus we decided to exclude infants younger than 2 years. We will also exclude any person who is terminally ill or any person with any known immunosuppressive condition, immune deficiency disease (including human immunodeficiency virus infection), or ongoing receipt of immunosuppressive therapy because of their increased risk of acquiring infections.

### Study Design and Procedures

**Figure 2:**
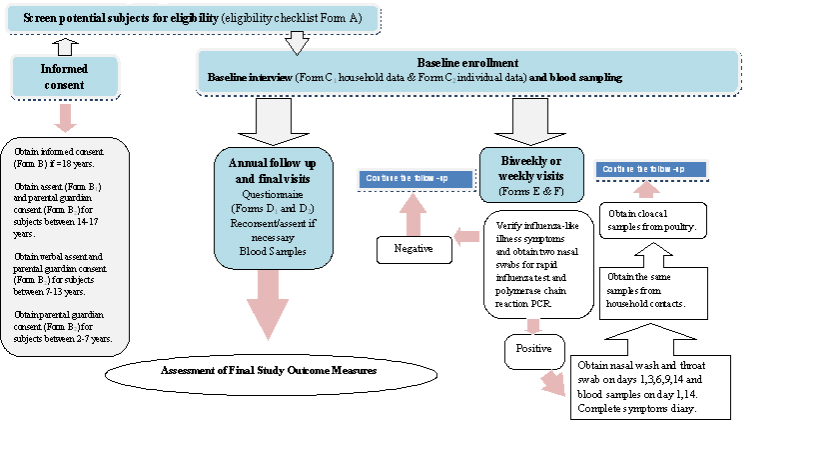
Schematic of the study design.

Figure 2 shows a schematic of the study design. We designed a 7-year prospective, household-based, cohort study to determine incidence and household transmission of AIV in humans exposed to poultry. At the start of the study, study staff will obtain informed consent, determine eligibility, and collect a serum sample to establish baseline levels of antibodies against AIV. Questionnaires specifically tailored for this study will be used (see Multimedia Appendix 1). Data on the demographics, health status, use of seasonal influenza vaccines, poultry exposure, and use of poultry influenza vaccines will be collected.

After enrollment, field nurses will visit each household twice weekly in the winter season (October through March) or weekly during the summer (April through September) to check if any subject has influenza-like illness symptoms (ILI). This schedule matches the increase in seasonal and AI activity in Egypt. Subjects (index cases) with confirmed ILI (ie, measured body temperature of > 38^o^C as well as cough and/or sore throat) will provide two nasal swabs for a point of care rapid influenza test and polymerase chain reaction (PCR). The nurse or physician will obtain nasal washes and throat swabs from subjects (index cases) testing positive for influenza A by rapid test or PCR on the nasal swab. The day on which a positive rapid test or PCR is obtained, will be day 1 of sampling. The nurse will also obtain 2x 3-milliliters (ml) blood samples, one for serology to test for antibodies against AIV, and another for isolating peripheral blood mononuclear cells (PBMC) on days 1 and 14. Furthermore, the nurse or physician will obtain nasal washes, throat swabs, and blood samples from all household contacts of the index case on days 1, 3, 6, 9, and 14. A symptoms diary will be started on day 1 and will continue to day 14. Field assistants trained to obtain swabs from poultry will collect cloacal swabs from the poultry in the household on day 1. Any household contact who reports ILI symptoms during the follow-up visits to the index case will then be followed up as per the same follow-up regime of the index case. Study subjects may get infected several times during the same season or over the course of the study.

Annually, all the study subjects will be interviewed again to note any changes in exposure variables. At this time, another blood sample will be obtained and tested for any changes in influenza-specific antibody level.

Human subjects’ approval will be sought from the Institutional Review Board of St Jude’s Children’s Research Hospital, Memphis, Tennessee (FWA0004775) and the Ethics Committee of the National Research Centre, Giza, Egypt (FWA00014747). The Institutional Animal Care and Use Committee of St Jude Children’s Research Hospital approved animal work.

### Sample Size Calculation

Our current data indicate that the seroprevalence of H5N1 among Egyptians exposed to poultry is around 2.1% (15/708); however, other experts estimate incidence rates to be around 0.5% [16]. Assuming that the infection rate in people exposed to H5N1 infected chickens is 0.5%, and that outcome in the unexposed population is 0.01%, and that the ratio of unexposed to exposed is equal to 9 (10% incidence in chickens), we estimate that 2134 people will be needed in our study to achieve 95% confidence and 80% power, to capture a difference (East 6). We plan to enroll 2400 poultry-exposed individuals, considering an approximate 10% lost to follow-up. If the sample seroprevalence of H5N1 is 1% or 2%, with about 10% of the participants lost to follow-up, the margin of error will be 0.4% or 0.6%, respectively. If the sample incidence rate is 0.5% or 0.25%, the margin of error will be 0.3% or 0.2%, respectively.

### Specimen Collection and Handling

The field nurse will collect a tube of blood (3 ml) in serum separator tubes. The blood will be allowed to clot at room temperature, and then kept on ice until it arrives at the laboratory on the same day as collection, where the specimens will be then centrifuged. Serum specimens will be aliquoted into multiple cryo-vials, labeled, and preserved at -20°C until ready for laboratory study. Swabs and nasal washes will be kept in tubes and sterile cups, respectively, containing viral transport medium and kept on ice until received in the laboratory, where they will be stored at -80°C until ready for laboratory study. Blood collected for PBMC isolation will be collected in tubes specific for this purpose, then kept on ice until it arrives at the laboratory on the same day as collection, where the specimens will be then processed, collected PBMCs will be preserved in liquid nitrogen until ready for laboratory analysis.

### Laboratory Analysis

Sera will be screened for human antibodies (IgG) against AIV H5N1 and H9N2 using a microneutralization assay at a dilution of 1:10 [17]. Viruses used in this assay will be matched to the viruses circulating in the poultry at the time of serum collection. Sera that screen positive will be further studied through a microneutralization assay procedure to determine full titer. Antibodies titer 1:80 and more will be considered positive; such high threshold of antibody titers met the criteria of The World Health Organization, and avoids cross reactivity that can result from anti-H3 and anti-H1 human influenza viruses. Due to the potential cross reactivity of antibodies against human influenza viruses, we will also evaluate the sera against recently circulating human influenza virus subtypes H1N1 and H3N2 using a hemagglutination inhibition assay [18].

Nasal swabs, oropharyngeal swabs, and nasal washes obtained from subjects reporting ILI symptoms and their household contacts will be screened for the presence of influenza A viruses by reverse transcriptase (RT) PCR amplifying the M gene. Further subtyping, culture, and sequencing will be conducted. PBMCs will be tested for innate and adaptive cell phenotyping by flow cytometry and functional assays that will be readout by flow cytometry and enzyme-linked immunofluorescence spot.

Cloacal swabs from poultry owned by subjects reporting ILI will be screened for the presence of influenza A viruses by RT PCR for the M gene. Further subtyping, culture, and sequencing will be conducted for identifying genetic and antigenic characteristics of AIV.

## Results

We will estimate new cases (including both index cases and infected household contacts) in poultry-exposed individuals by detecting influenza viruses in throat and nasal swabs and nasal washes, obtained from subjects, using rapid tests and molecular techniques. As defined earlier, the overall incidence will be estimated as the proportion of all new cases identified among all study subjects per influenza season or per year. A 95% confidence interval will be provided.

We will measure antibodies against AI viruses in sera collected from all poultry-exposed individuals no matter whether they have confirmed influenza or not. The overall seroprevalence will be estimated as the proportion of sero-positive subjects among all study subjects per influenza season or per year. A 95% confidence interval will be provided.

We will use a questionnaire that collects specific occupational, environmental, and behavioral risk factors. Because we expect both the overall incidence and seroprevalence to be very low, descriptive statistics (frequencies, proportions, etc) stratified by potential risk factors will be summarized to briefly describe the potential trend.

For determining the human-to-human transmission rate, only secondary infections will be considered and analyzed. We will obtain throat and nasal swabs, blood, and nasal washes from the household contacts of poultry-exposed individuals with a confirmed influenza A infection and test them for the presence of influenza A viruses or antibodies against influenza A viruses. We assume that if an illness developed in a household contact of an index patient, the household contact is infected with the virus from the index patient. As defined earlier, the secondary infection rate will be estimated as the proportion of susceptible household contacts of identified index cases with confirmed influenza infection among all susceptible household contacts of identified index cases per influenza season or per year. A 95% confidence interval will be provided.

## Discussion

### Household Transmission Study

Our previous study about the seroprevalence of H5N1 infection in Egyptians exposed to poultry showed that the number of reported cases is greatly underestimated and that the case-fatality rate is consequently greatly overestimated. However, even the most accurate measurement of seroprevalence cannot indicate the true extent of human infection with H5N1 viruses, as we know too little about the factors that influence the timing and likelihood of seroconversion after exposure. By conducting a large-scale prospective household-based study, we will be able to study the immune response that pursues after infection, and we will be able to characterize the AIV causing infection and compare them to those circulating among the poultry.

This study may have several limitations. Because we will use convenience sampling, selection bias may affect our results. However, poultry backyard-raising practices in rural Egypt are generally homogenous, thus limiting the effects of selection bias on conclusions drawn from this study. Misclassification bias can occur in case of underestimated poultry exposure.

### Conclusions

Data from this study may yield a better understanding of incidence and potential risk factors for infection with AIV in poultry-exposed individuals and secondary infection risk for household contacts. The data may well also assist in developing an understanding of the relationship of virus strains to disease severity. The data can estimate AI burden in exposed humans and help decision makers prioritize resources and plan public health interventions. Coupled with results from active AIV surveillance among Egyptian poultry, our findings would provide a better image of the current AIV situation in the country.

## Appendix

Multimedia Appendix 1 Household questionnaire.

## Abbreviations

AI: avian influenza
AIV: avian influenza viruses
ILI: influenza-like illness symptoms
ml: milliliters
PBMC: peripheral blood mononuclear cells
PCR: polymerase chain reaction
