# Supplementary material for: Household Transmission of Zoonotic Influenza Viruses in a Cohort of Egyptian Poultry Growers
Source: JMIR Res Protoc. 2015 Jun 22;4(2):e74. doi: 10.2196/resprot.4331 (PMC4526956; doi:10.2196/resprot.4331)
Supplement: Multimedia Appendix 1 [file resprot_v4i2e74_app1.pdf]

Today's date:

Day month year

Location ID:

Insert Barcode:

FORM C 1 *Household data*

Address

Governorate **Village**

GPS East

GPS North

Phone numbers:

Mobile Work

Home Others

Today's date:

Day month year

Location ID:

1- How many individuals live in your house?

Family pedigree

Head of the house

Today's date:

Day month year

Location ID:

male

femal
e

Today's date:

Day month year

Location ID:

2-What is your monthly expenditure on food per month?

- ○ 501-1000
- ○ 1001-1500
- ○ >1500
- ○ Unknown

Today's date:

☐☐ ☐☐ ☐☐

Day month year

Location ID:

3- Identify the animals from the list below that you currently have in your flock:

	Yes	No	Number of animals
**Chickens**			
**Ducks**			
**Geese**			
**Pigeons**			
**Turkey**			
**Rabbits**			
**Cows**			
**Buffaloes**			
**Sheep**			
**Donkeys**			
**Horses**			
**Goats**			
**Dogs**			
**Cats**			
**Camels**			

Today's date:

Day month year

Location ID:

Today's date:

☐☐ ☐☐ ☐☐

Day month year

Location ID:

4-Where do you usually keep your poultry?

- ○ **In a barn**
- ○ **On the roof**
- ○ **Inside the house**

5-What do you do if you find dead poultry?

- ○ **Incinerate**
- ○ **Bury**
- ○ **Throw it in the trash**
- ○ **Throw it in water stream**
- ○ **Get rid of it in a closed bag in the trash**

6- What do you do if you suspect diseased poultry?

- ○ **Separate it from other animals**
- ○ **Slaughter it**
- ○ **Seek medical advice**
- ○ **Get rid of it**
- ○ **Do nothing**

Today's date:

Day	month	year

Location ID:

Poultry vaccination

7-Do you vaccinate your poultry against avian influenza?

○ **Yes** ○ **No**

8- What type of vaccine do you use?

○ **H5N1** ○ **H9N2**

○ **Other** ○ **Unknown**

9-Who administers the vaccine?

○ **Veterinarian** ○ **Worker**

○ **Family member**

10- When was the last vaccine administered on your flock?

Day	Month	Year

Today's date:

Day month year

Location ID:

For investigator use only

Was Form reviewed ○ ○ yes No

I certify that the information recorded here is complete and has been accurately recorded:

Initials

Date Day Month [illegible]

General comments
